# Editorial: Mechanisms by Which SLE-Associated Genetic Variants Contribute to SLE Pathogenesis

**DOI:** 10.3389/fimmu.2019.02808

**Published:** 2019-12-03

**Authors:** José C. Crispín, Laurence Morel

**Affiliations:** ^1^Department of Immunology and Rheumatology, Instituto Nacional de Ciencias Médicas y Nutrición Salvador Zubirán, Mexico City, Mexico; ^2^Tecnológico de Monterrey, Escuela de Medicina y Ciencias de la Salud, Mexico City, Mexico; ^3^Department of Pathology, Immunology, and Laboratory Medicine, University of Florida, Gainesville, FL, United States

**Keywords:** lupus (SLE), SNP, genetic variant, pathogenesis, autoimmunity

Systemic lupus erythematosus (SLE) is a complex disease strongly influenced by genetic factors (1). Through their effects on gene expression and function, genetic variants may modify disease manifestation and outcomes by facilitating certain cellular behaviors (2). This Research Topic brings together original and review papers that explore how individual genes and their variants may affect SLE development, pathogenesis, and therapeutics. Alperin et al. present a review article that describes monogenic syndromes that share clinical and pathological similarities with SLE. The extreme phenotypes associated with the genetic deficiencies that cause these syndromes demonstrate the role of individual genes in the immune system. Martínez-Bueno and Alarcón-Riquelme present a bioinformatics imputation analysis that identifies 98 candidate genes that may contribute to SLE through rare variants that cannot be detected in conventional genetic association studies.

Transcription of the long non-coding RNA Linc00513 is shown by Xue et al. to be affected by an SLE-associated single-nucleotide polymorphism (SNP). Cells bearing the risk allele have increased levels of Linc00513 because the SLE-associated variant promotes its transcription in response to type I IFN. This lncRNA facilitates the expression of a relatively large number of IFN-induced genes. Therefore, the presence of the risk allele could amplify the signal conveyed by type I IFN.

The paper by Ju et al. describes a previously unknown variant of the *Nasp* gene identified as a pathogenic element located in the *Slec1* sublocus of the NZM2410 mouse (3, 4). The lupus-associated variant modified the sNASP protein resulting in an increased capacity to bind histones. Importantly, in the presence of the lpr mutation (Fas^*lpr*^), the risk variant of *Nasp* increased lymphoproliferation and tissue inflammation (lung and kidney), suggesting that it may possess a pathogenic capacity.

Gorman et al. present a thorough analysis of the functional effects of the *TYK2*^P1104A^ variant that protects against multiple autoimmune diseases, including SLE (5). They show that healthy humans carrying the protective allele have a lower number of circulating follicular helper T cells (T_FH_) and switched memory B cells. Moreover, the amino acid substitution decreased the response of CD4 T cells to IL-12, IL-23, and IFN-α, confirming that it represents a hypomorphic allele. *In vivo*, the *Tyk2*^P1104A^ variant protected mice from experimental autoimmune encephalomyelitis (EAE), although it did not show any effects in two models of murine lupus-like disease.

Molineros et al. conducted a detailed study that identified a SNP (rs11631591) that facilitates binding of hnRNP-K. Because it is located in an enhancer region, the risk allele increases the expression of *RASGRP1* and, consequently, MAP kinase signaling. Calcium/Calmodulin Kinase IV (CaMK4) is a serine/threonine kinase that regulates cell signaling and gene expression in a variety of cells that includes T cells, podocytes, and mesangial cells. Expression levels and activity of CaMK4 are abnormally increased in T cells from patients with SLE (6) and in renal cells in a variety of immune and non-immune conditions (7). The review by Ferretti et al. describes the role of CaMK4 in human disease and mouse models and discusses strategies to block the activity of this kinase as interesting and novel therapies.

Two of the papers explore the effects that sex, either through chromosomal or hormonal differences, imposes on gene expression and function (Harris et al.) and disease phenotype (Savelli et al.). Finally, the review by Vukelic et al. discusses novel therapeutic strategies in SLE.

The papers included in this Research Topic illustrate the complex relationship between genetic variants, environmental stimuli, and immune function, and offer a glance into how individual variants may affect the behavior of specific types of cells in manners that may promote or avoid autoimmune and/or inflammatory organ damage. We believe that understanding how genetic variants affect immune function in the steady state and in the setting of chronic inflammation will improve our capacity to predict disease phenotypes, including prognosis and response to therapy of individual patients.

## Author Contributions

JC and LM: conceptualization and writing.

### Conflict of Interest

The authors declare that the research was conducted in the absence of any commercial or financial relationships that could be construed as a potential conflict of interest.
